# Individual and Neighborhood Level Predictors of Children’s Exposure to Residential Greenspace

**DOI:** 10.1007/s11524-024-00829-z

**Published:** 2024-03-14

**Authors:** Marnie F. Hazlehurst, Anjum Hajat, Adam A. Szpiro, Pooja S. Tandon, Joel D. Kaufman, Christine T. Loftus, Nicole R. Bush, Kaja Z. LeWinn, Marion E. Hare, Sheela Sathyanarayana, Catherine J. Karr

**Affiliations:** 1 Department of Environmental & Occupational Health Sciences, University of Washington School of Public Health, Seattle, WA USA; 2 Department of Epidemiology, University of Washington School of Public Health, Seattle, WA USA; 3 Department of Biostatistics, University of Washington School of Public Health, Seattle, WA USA; 4 Seattle Children’s Research Institute, Seattle, WA USA; 5 Department of Pediatrics, School of Medicine, University of Washington, Seattle, WA USA; 6 Division of General Internal Medicine, Department of Medicine, University of Washington School of Medicine, Seattle, WA USA; 7 Department of Pediatrics, School of Medicine, University of California, San Francisco, CA USA; 8 Department of Psychiatry and Behavioral Sciences, School of Medicine, University of California, San Francisco, CA USA; 9 Department of Preventive Medicine, University of Tennessee Health Science Center, Memphis, TN USA

**Keywords:** Childhood opportunity index, Weighted quantile sum regression, Greenspace, Racial residential segregation, Pediatric

## Abstract

Inequities in urban greenspace have been identified, though patterns by race and socioeconomic status vary across US settings. We estimated the magnitude of the relationship between a broad mixture of neighborhood-level factors and residential greenspace using weighted quantile sum (WQS) regression, and compared predictive models of greenspace using only neighborhood-level, only individual-level, or multi-level predictors. Greenspace measures included the Normalized Difference Vegetation Index (NDVI), tree canopy, and proximity of the nearest park, for residential locations in Shelby County, Tennessee of children in the CANDLE cohort. Neighborhood measures include socioeconomic and education resources, as well as racial composition and racial residential segregation. In this sample of 1012 mother–child dyads, neighborhood factors were associated with higher NDVI and tree canopy (0.021 unit higher NDVI [95% CI: 0.014, 0.028] per quintile increase in WQS index); homeownership rate, proximity of and enrollment at early childhood education centers, and racial composition, were highly weighted in the WQS index. In models constrained in the opposite direction (0.028 unit lower NDVI [95% CI: − 0.036, − 0.020]), high school graduation rate and teacher experience were highly weighted. In prediction models, adding individual-level predictors to the suite of neighborhood characteristics did not meaningfully improve prediction accuracy for greenspace measures. Our findings highlight disparities in greenspace for families by neighborhood socioeconomic and early education factors, and by race, suggesting several neighborhood indicators for consideration both as potential confounders in studies of greenspace and pediatric health as well as in the development of policies and programs to improve equity in greenspace access.

**Supplementary Information:**

The online version contains supplementary material available at 10.1007/s11524-024-00829-z.

## Introduction

Higher greenspace exposures have been linked to an array of beneficial physical and mental health outcomes across the life course [1–3]. Greenspace in urban areas may promote healthy behaviors such as physical activity, mitigate environmental risk factors such as heat, and restore cognitive and psychological capacities [4]. Despite growing evidence for greenspace as a health resource, inequities in greenspace exposures exist in many urban settings [5]. Differential availability of and access to greenspace in residential neighborhoods in the US by sociodemographic factors, such as race, ethnicity, and socioeconomic status, has been shaped by the history of urban development and biased policies favoring some groups. Current greenspace and tree canopy levels have been linked to discriminatory housing policies, such as redlining, racial covenants, and zoning regulations [6–10]. Structural racism—including these policies contributing to racialized residential segregation and the subsequent disinvestment of resources in these communities—has influenced the current structure of built and green urban environments [11–13].

However, specific patterns of greenspace by race and socioeconomic status are not consistent across all urban settings or for all measures of greenspace [14–16]. For example, different patterns of greenspace availability, including surrounding greenness or tree cover, have been identified compared to measures of park proximity [17, 18]. While prior studies have primarily investigated neighborhood income or racial composition in relation to greenspace, few have examined how greenspace relates to a broader set of neighborhood conditions or incorporated spatial measures of racial residential segregation. Additionally, averaging greenspace over administrative zones such as census tracts may obscure important variation in exposures to greenspace; finer spatial scales for greenspace exposures have been identified as important to improving estimation of health effects [19]. Individual and neighborhood level socioeconomic factors are not perfectly correlated and information at both levels more fully describes an individual’s living circumstances than data from only one level [20]. Individual-level factors may influence residential selection into a location within a neighborhood and predict greenspace in close proximity to an individual’s residential location or at residences near administrative boundaries, but prior ecologic studies have largely focused only on neighborhood or city-wide data. Furthermore, understanding relationships at multiple levels has implications for addressing confounding in analyses of greenspace health effects.

In this study, we examined relationships of neighborhood and individual-level factors with surrounding greenness, tree canopy, and park proximity, within a cohort of mother–child dyads in Tennessee. We leveraged multiple statistical methods to explore the connections between a large suite of neighborhood factors-including indicators of socioeconomic status, other neighborhood resources for families, and racial residential segregation-and greenspace at locations where children live. First, we estimated the overall association between the mixture of neighborhood factors and greenspace. Second, we evaluated whether neighborhood-level, individual-level, or the combination of these factors best predicted greenspace measures.

## Methods

### Study Population

We used data from the Conditions Affecting Neurocognitive Development and Learning in Early childhood (CANDLE) study, one of three pregnancy cohorts within the ECHO-PATHWAYS Consortium [21–23]. CANDLE is a sociodemographically diverse cohort that was established specifically to investigate determinants of child neurodevelopment among a population-based sample of mother–child dyads in Shelby County, Tennessee. Women were enrolled in CANDLE during mid-pregnancy (*n* = 1503 in 2006–2011) and follow-up was conducted throughout childhood, including a study visit when children were 4–6 years old in 2011–2016. Analyses were limited to those participants who attended the age 4–6 follow-up visit and reported a current address within Shelby County that could be validly geocoded to an exact latitude/longitude (e.g., parcel with match score ≥ 93, or exact street address or nearest intersection with match score ≥ 85), which was linked to the greenspace measures and neighborhood factors.

CANDLE research activities were approved by the University of Tennessee Health Sciences Center Institutional Review Board (IRB). The analyses included here were conducted as part of the ECHO-PATHWAYS Consortium, approved by the University of Washington IRB.

### Residential Greenspace

Greenspace was operationalized in three ways, as (1) residential surrounding greenness, (2) tree canopy, and (3) proximity of the nearest park.

#### Surrounding Greenness

Surrounding greenness was defined using NDVI, calculated from the NASA Global Web-Enabled Landsat Data (GWELD) v031 2011 30-m resolution annual dataset [24]. This time frame was selected due to its proximity to address ascertainment in this cohort and based on data availability. Satellite imagery was calibrated using top-of-atmosphere reflectance. The maximum NDVI value obtained during the annual 2011 sampling timeframe, which tended to be during summer months, was selected for each pixel (possible range: − 1 to 1). Healthy vegetation, with chlorophyll in plant leaves absorbing visible light for photosynthesis, results in an NDVI closer to 1, whereas impervious surfaces yield an NDVI closer to zero. Pixels less than zero, indicating water, were set to missing. The average of non-missing values was calculated within a 300-m radial buffer of the participant address, a commonly utilized distance measure of the residential neighborhood in policy and programs, and hypothesized to be a relevant scale for psychological mechanisms in health effects analyses [19, 25]. Buffers of 100 m and 1000 m were explored in sensitivity analyses.

#### Tree Cover

Tree cover was calculated as the percent of land covered by trees within the census block group in which the participant address was located, using data obtained from the US Environmental Protection Agency EnviroAtlas Memphis Community Dataset [26]. Tree coverage was derived from 1-m resolution land cover data and includes street trees, trees in park areas, urban forests, and single trees on various properties.

#### Park Proximity

Park proximity was defined as the Euclidean distance from the residential location to the edge of the nearest public park. Park boundaries were derived from ParkServe data compiled by the Trust for Public Land, which includes publicly owned local, state, and national parks; school parks with a joint-use agreement with local government; and privately owned parks that are managed for full public use [27]. Home Owner Association parks, golf courses, and cemeteries were not included.

### Neighborhood Factors

#### Childhood Opportunity Index

Neighborhood conditions were conceptualized as both socioeconomic and educational resources in the neighborhood, using the publicly available Childhood Opportunity Index v2 (COI). The COI was developed based on empirical evidence and conceptual frameworks to examine neighborhood resources and conditions that influence children’s health and development [28, 29]. See details elsewhere [30, 31]. Briefly, the COI was compiled from multiple data sources within the domains of socioeconomic resources and educational opportunity in order to capture a variety of potential resources including the availability and quality of neighborhood institutions and neighborhood social structure and economic resources [30, 31]. Some variables were included in the COI because they reflect the broader learning environment for children in the neighborhood, including educational opportunities both in early childhood and at school-age, such as access to libraries, and afterschool and community programs. Other variables reflect financial resources that may fund neighborhood programs and amenities. All variables in the COI were selected based on relevance for child health and healthy development. Data from 2010 at the census tract level were used to calculate *z*-scores for each variable.

#### Racial Residential Segregation

We used measures of both racial composition within the census tract and a spatial measure accounting for neighboring tracts as a proxy for and marker of racial residential segregation. Racial composition measures (percentage Black residents and percentage White residents) were obtained for each census tract from the 2006–2011 American Community Survey (ACS). Consistent with much of the prior literature, the percentage of Black residents in the neighborhood is considered as a proxy for exposure to the forms of structural racism that people experience resulting from residential segregation [12]. To further assess spatial patterns of segregation in this study, we used the Getis-Ord G_i_* statistic (*G* statistic) [32–34]. The clustering dimension of racial residential segregation assessed by the *G* statistic has been theorized to be a relevant measure for understanding historical patterns of land-use decision-making and community investment in physical infrastructure [35]. This statistic, calculated using racial composition data from the 2006–2011 ACS, yields a *z*-score that describes whether the racial composition within the census tract and in the surrounding tracts (defined as those that share a boundary) deviates from the overall county mean. In this study, a higher positive value of the *G* statistic indicates an overrepresentation of Black residents within the census tract and surrounding tracts compared to Shelby County as a whole. This spatial measure of racial residential segregation addresses two limitations of aspatial measures of segregation; aspatial measures do not account for the spatial proximity of neighborhoods or the composition of adjacent census tracts, and administrative boundaries may not reflect the local environments of those within the census tract especially for those living near the boundary of a tract.

#### Urbanicity

Urbanicity was assessed as population density at the census tract level, derived from the 2006–2011 ACS. Urbanicity was considered as a confounder because Shelby County includes both urban and rural census tracts, it predicts greenspace availability, and it is correlated with measures of neighborhood resources.

### Individual and Household Characteristics

Individual characteristics included maternal age, education (less than a high school degree, high school diploma or equivalent, technical school, college degree, and graduate or professional degree), and marital status (married/living with a partner or single); in addition, mothers reported on their self-identified race (reported as African-American/Black, White, Asian, American Indian/Alaska Native, Native Hawaiian/Pacific Islander, or another race), which was later collapsed into 3 categories of African-American/Black, White, or all other self-identified races. Household characteristics included household income and household size. Income was reported in 11 categories; the midpoint of each category (or $80,000 for those in the highest group) was selected and treated as a continuous variable.

### Statistical Analyses

All analyses were conducted in R 3.6 [36]. Descriptive statistics and Spearman correlations were used to examine the distribution of and correlations between the greenspace measures and each of the predictors. An overview of potential analytic approaches and rationale for selecting the following methods is included in Appendix A.

#### WQS Regression

Weighted quantile sum (WQS) regression was developed in the context of studies of chemical mixtures [37, 38], but has been extended to consider socioeconomic variables [39, 40]. WQS was selected because it performs well with correlated predictors and the model can be constrained in the positive or negative direction to investigate mixtures in which components may have separate directional associations with the outcome. In this study, WQS regression was used to estimate the association between a suite of neighborhood factors and greenspace, and to examine which predictors were driving those associations. Neighborhood conditions (*n* = 19) derived from the COI socioeconomic and educational opportunity scales, and racial composition and spatial residential segregation measures, were included in the WQS index. Variables were reverse scored as necessary and divided into quintiles, such that for all variables higher quintiles represent more neighborhood resources, higher socioeconomic status, or lower exposure to residential segregation, and were hypothesized to be associated with more greenspace compared to lower quintiles.

WQS regression includes a two-step process. First, a likelihood-based model is optimized, subject to bootstrap resampling of observations to stabilize the optimization, to select weights for quantile versions of the independent variables in a WQS index of the form ${\text{WQS}}={\sum }_{j=1}^{c=19}{w}_{j}{q}_{ij}$ where ${w}_{j}$ is the weight ($0\le {w}_{j}\le 1$) for the *j* variable with quantile values ${q}_{j}$, and the weights sum to 1. The weights are optimized to identify the strongest association in a prespecified direction between the WQS index and the dependent variable in an adjusted linear regression model.

In the second step, the WQS index is included in an ordinary least squares regression model that adjusted for urbanicity. For each measure of greenspace as the dependent variable, the coefficient of the WQS index, which describes the relationship between neighborhood factors and residential greenspace, was estimated. *p* values for WQS regression in the full sample are known to be anti-conservative and therefore we used a permutation test to calculate *p* values (*p*_permutation_) for each model [41]. Two separate models were calculated by constraining the association between the WQS index and the greenspace measure to be either positive or negative. We hypothesized that a higher WQS index would be associated with higher levels of surrounding greenness and tree canopy and closer proximity to parks. The weights in the WQS index were examined to identify the factors driving observed associations.

The gWQS R package (version 3.0.4; https://cran.r-project.org/web/packages/gWQS) was used to run the WQS models and the wqspt package (version 1.0.1; https://cran.r-project.org/web/packages/wqspt) was used for permutation tests.

#### LASSO Regression

Least Absolute Shrinkage and Selection Operator (LASSO) regression models were used to compare prediction of greenspace using predictors at the neighborhood level (Model A), individual level (Model B), or from both levels in the same model (Model C). Predictors in these models included 21 neighborhood factors, including measures of socioeconomic and education resources, and racial residential segregation, as well as six individual-level characteristics. Continuous predictors were standardized to mean 0 (SD 1). The minimum mean squared error (MSE) from tenfold cross-validation was compared across Models A–C to assess the predictive ability of the three sets of variables. Coefficients for predictors in each model were then estimated in the full sample using the corresponding tuning parameter.

## Results

This analysis included 1012 mother–child dyads living in 203 of the 221 census tracts in Shelby County (Table 1, Supplemental Fig. 1). LASSO models were further restricted to those with complete data for individual-level predictors (*n* = 917). In this sample, 63% of women self-identified as African-American/Black, 30% as White, and 6% as another race reported in categories that were combined due to small subsample size. The mean annual household income was $37,700 (SD 27,900), and 41% of participants had a college degree. Participants were sampled from Shelby County, and the cohort was generally representative of the demographics of Shelby County overall based on race, education, and income.

**Table 1:** Sample characteristics

	CANDLE cohort (*N* = 1012) ^a^			
**Individual/household level**	Mean (SD) or *N* (%)	25th p	Median	75th p
Maternal age (years), mean (SD)	31.4 (5.4)	26.8	30.9	35.4
Maternal race, *n* (%)				
Black/African-American	621 (63%)	-	-	-
White	298 (30%)	-	-	-
Another race	63 (6%)	-	-	-
Maternal education, *n* (%)				
Less than high school	48 (5%)	-	-	-
GED or high school diploma	401 (41%)	-	-	-
Technical school	121 (12%)	-	-	-
College degree	246 (25%)	-	-	-
Graduate or professional degree	159 (16%)	-	-	-
Marital status, *n* (%)				
Married/living as married	562 (58%)	-	-	-
Single/living as single	415 (42%)	-	-	-
Annual household income ($), mean (SD) ^b^	37,700 (27,900)	12,500	30,000	60,000
Household size, mean (SD)	4.5 (1.4)	4.0	4.0	5.0
**Neighborhood level **^c^	Mean (SD)	25th p	Median	75th p
*COI socioeconomic domain*^d^				
Poverty rate (% households)	22.9 (16.3)	7.4	19.4	35.5
Public assistance rate (% households)	23.5 (16.4)	9.1	22.7	37.6
Homeownership rate (%)	58.1 (21.6)	41.9	60.5	74.7
High-skill employment (%)	29.5 (16.1)	16.1	26.2	41.3
Median household income ($)	50,853 (29,184)	29,397	41,059	67,288
Single-headed households (%)	55.0 (25.1)	34.2	57.8	76.4
Employment rate (%)	71.8 (12.6)	64.2	75.3	81.0
Commute duration (% commuting > 1 h one way)	3.1 (2.5)	1.3	2.5	4.6
*COI education domain*^d^				
School poverty (% of students)	71.1 (25.0)	56.7	78.6	91.6
Teacher experience (% in 1st or 2nd year)	7.5 (5.6)	4.2	5.9	9.0
Adult educational attainment (% w/ college degree)	24.2 (18.6)	8.6	17.3	37.8
Early childhood education centers (*n* in 5-miles)	4.9 (0.7)	4.6	5.1	5.4
High-quality early childhood education centers (*n*)	1.1 (2.9)	1.1	1.8	2.3
Early childhood education enrollment (%)	45.9 (24.8)	28.3	43.3	66.7
Third grade math proficiency^e^	157.4 (72.8)	105.3	125.8	194.5
Third grade reading proficiency^e^	145.5 (77.9)	89.1	113.8	186.1
High school graduation rate (%)	76.1 (9.9)	69.8	76.2	82.3
Advanced placement course enrollment (ratio)	0.12 (0.09)	0.1	0.1	0.2
College enrollment in nearby institutions (% in 25 miles)	36.0 (2.6)	33.7	36.0	37.8
*Racial composition and residential segregation* ^f^				
Proportion of Black/African-American residents	57.0 (33.9)	25.0	68.0	89.0
Proportion of White residents	36.9 (32.1)	9.0	22.0	67.0
Residential segregation *G* statistic	0.13 (1.87)	− 1.87	0.80	1.85

^a^Measures are shown from the age 4–6 year study visit. Missing maternal age (11), maternal education (7), marital status (5), household income (42), and household size (65)

^b^Household income was reported in US dollars in the following 11 categories: 0–4999, 5000–9999, 10,000–14,999, 15,000–19,999, 20,000–24,999, 25,000–34,999, 35,000–44,999, 45,000–55,999, 55,000–64,999, 65,000–74,999, or ≥ 75,000. The midpoint of each category was selected (or $80,000 for the highest group) and treated as a continuous variable

^c^Neighborhood is defined as the census tract of the address reported by participants at the time of the age 4–6 study visit

^d^Variables in the education and socioeconomic domains were obtained from the Childhood Opportunity Index (COI)

^f^3^rd^-grade math and reading proficiency reported as National Assessment of Educational Progress (NAEP) scale points

^e^Data on race at the census tract level was obtained from the American Community Survey 2006–2011. Residential segregation calculated as the *G* statistic

Among the census tracts in which CANDLE participants lived, the mean proportion of Black residents in the census tract was 57% (SD 34%), the mean proportion of White residents in the census tract was 37% (SD 32%), and the mean proportion of residents in additional groups was 6% (SD 5%). CANDLE participants on average tended to live in neighborhoods with a higher spatial dissimilarity measure of residential segregation. Both the proportion of Black residents in the census tract and the spatial residential segregation measure were generally negatively correlated with socioeconomic and education neighborhood opportunity measures (Supplemental Fig. 2). Relative to other US cities, neighborhoods in Memphis on average tend to have fewer neighborhood-level resources as assessed by the various components of the COI and larger gaps within the county between the neighborhoods with many opportunities and those with few resources [31].

**Fig. 1:**
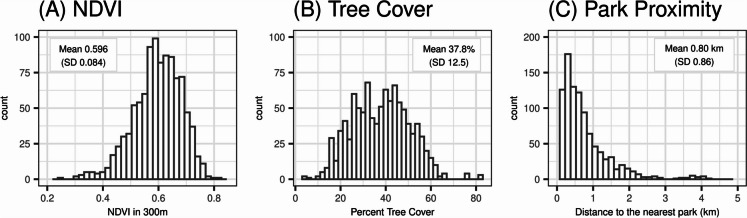
Distribution of (**A**) residential surrounding greenness, (**B**) tree cover, and (**C**) park proximity, in the CANDLE cohort for the residential address reported at the time of the age 4–6 year study visit (*n* = 1012). Residential surrounding greenness is assessed using the normalized difference vegetation index (NDVI) within a 300-m radial buffer of the home address. Tree cover is assessed as the percentage of the census block group. Park proximity is assessed as the distance to the nearest boundary of a park; the *x*-axis is truncated at 5 km for visualization purposes

NDVI (mean 0.596 (SD 0.084) in 300 m) and tree canopy levels (mean 37.8% (SD 12.5%) of census block group) were generally high in this sample (Fig. 1). While NDVI and tree canopy were correlated at 0.59, park proximity was not highly correlated with the other greenspace measures (Spearman correlations < 0.1) and 28% of participants lived within 300 m of the nearest park.

### WQS Regression

**Fig. 2:**
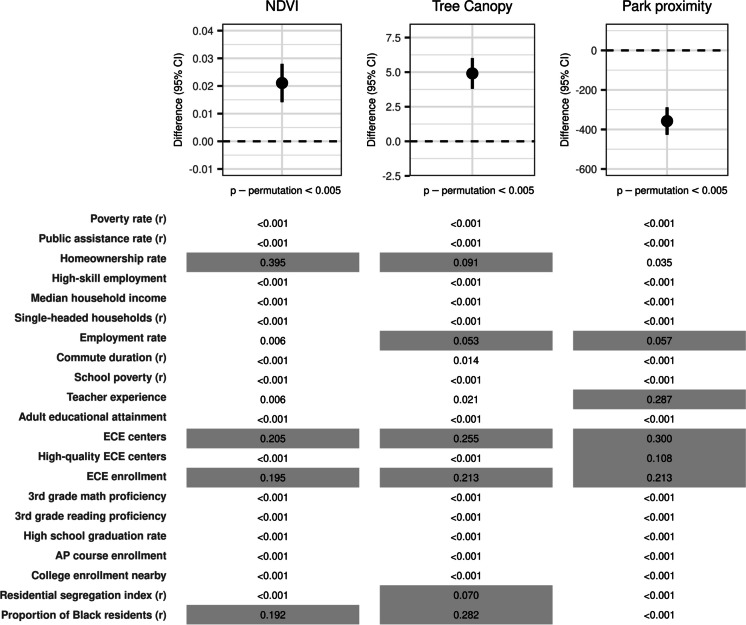
Association of neighborhood factors with residential surrounding greenness, tree cover, and park proximity in WQS regression constrained in the hypothesized direction. Plots show the difference and 95% confidence interval in NDVI, tree canopy, and park proximity per 1 quintile higher WQS index, with the *p* value from the permutation test displayed at the bottom of each plot. The corresponding weights for each variable in the WQS index are shown underneath the respective plot. Some variables, indicated by (*r*), were reverse coded before being considered in the WQS index such that for all independent variables a higher value was hypothesized to be associated with more greenspace. Weights are shaded gray where the value of the weight is greater than it would be if weights were distributed equally across all variables in the index (i.e., gray shading indicates weight > 0.048). A 1 quintile increase in the WQS index was associated with a 0.021 unit higher NDVI (95% CI: 0.014, 0.028) and this association was largely driven by homeownership rate, early childhood education (ECE) centers and enrollment, and proportion of Black residents in the census tract. A 1 quintile higher WQS index was associated with a 4.9% higher tree canopy coverage (95% CI: 3.8, 6.0) and this index appears similar to that for NDVI, with proportion of Black residents in the census tract, ECE centers and enrollment, and homeownership rate weighted most heavily. A 1 quintile higher WQS index was associated with living 358 m closer to a park (95% CI: − 427, − 288) and the early childhood education variables were weighted heavily, along with teacher experience

In our primary analysis (Fig. 2), a 1 quintile increase in the WQS index was associated with a 0.021 unit higher NDVI (95% CI: 0.014, 0.028; *p*_permutation_ < 0.005), approximately 25% of the SD. Higher homeownership rate, closer proximity of early childhood education (ECE) centers and higher enrollment, and lower percentage of Black residents were highly weighted. The magnitude of the estimated coefficient for the WQS index of 0.021 corresponds to approximately a quarter of the standard deviation in the NDVI measure (SD 0.084) and was small relative to the overall mean NDVI in this sample. A 1 quintile higher WQS index was associated with a 4.9 percent higher tree canopy coverage (95% CI: 3.8, 6.0; *p*_permutation_ < 0.005), and the relative weights are similar to that for NDVI, with the same four variables having the highest weights. A 1 quintile higher WQS index was associated with living 358 m closer to a park (95% CI: − 427, − 288; *p*_permutation_ < 0.005), and the ECE variables were weighted heavily, along with teacher experience.

**Fig. 3:**
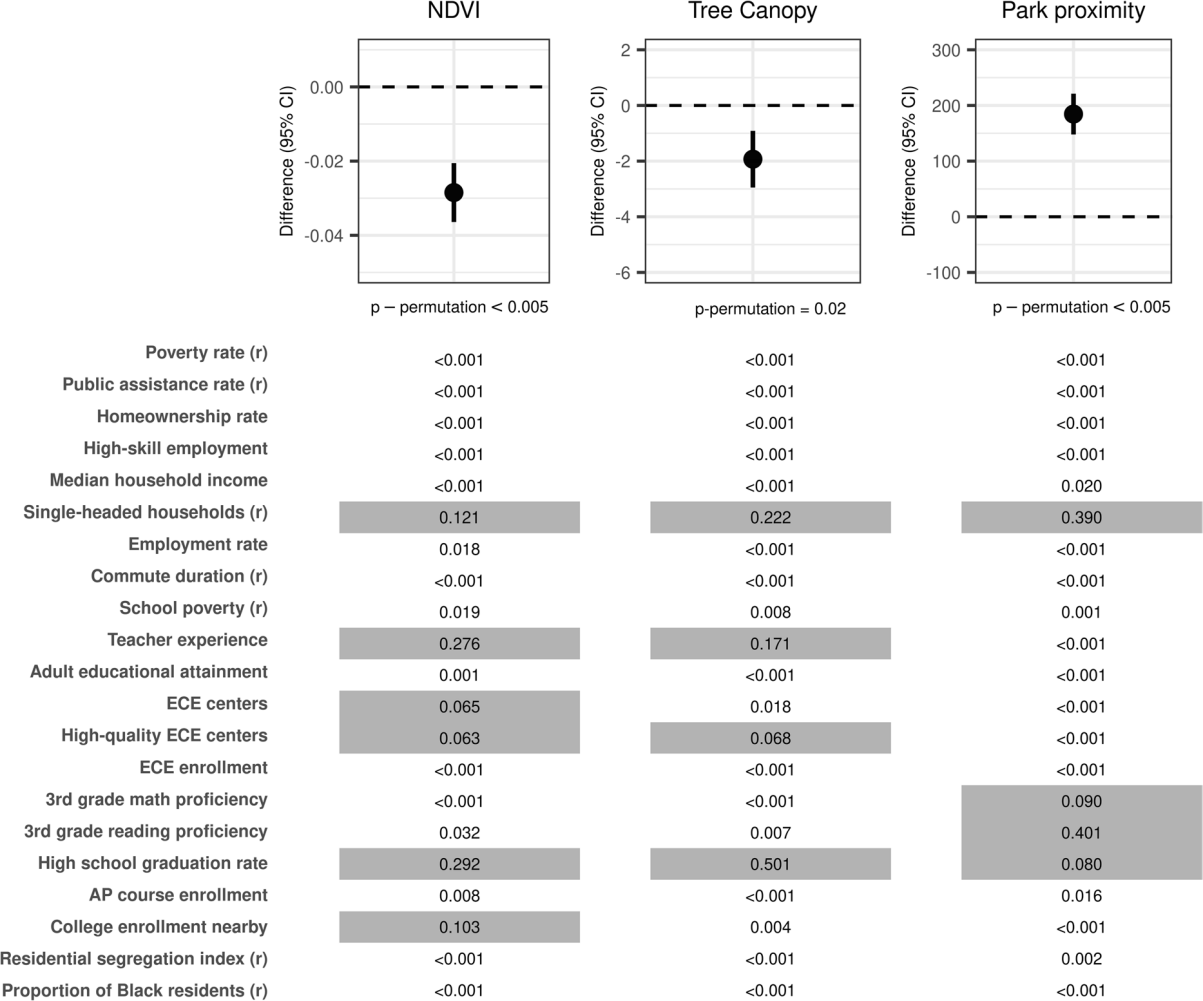
Results from the WQS regression with association between neighborhood factors and NDVI, tree canopy, and park proximity constrained in the opposite of hypothesized direction. Plots show the difference (95% confidence interval) in surrounding greenspace per 1 quintile higher WQS index in a model where the association is constrained in the opposite of the hypothesized direction, with the *p* value for the permutation test indicated under the plot. Weights greater than 0.048, which indicates those variables weighted higher than they would be if weights were distributed equally across all variables in the index, are shown in gray. Some variables in the WQS index were reverse coded, indicated by (*r*), so that variables contributing to the WQS index were coded such that a higher value of the variable was hypothesized to be associated with more greenspace. A 1 quintile higher WQS index was associated with 0.028 lower NDVI (95% CI: − 0.036, − 0.021; *p*_permutation_ < 0.005) with high school graduation rate and teacher experience highly weighted. A 1 quintile higher WQS index was associated with 1.9 lower percent tree canopy (95% CI: − 3.0, − 0.9; *p*_permutation_ = 0.02), with high school graduation rate highly weighted. A 1 quintile higher WQS index was associated with a 184-m further distance to the nearest park (95% CI: 148, 221; *p*_permutation_ < 0.005) with 3rd-grade reading proficiency and single-headed households (reverse coded) highly weighted

In models constrained in the direction opposite of that hypothesized, statistically significant associations (*p*_permutation_ < 0.05) were observed for all three greenspace measures (Fig. 3). A 1 quintile higher WQS index was associated with a 0.028 unit lower NDVI (95% CI: − 0.036, − 0.020; *p*_permutation_ < 0.005), and high school graduation rate, teacher experience, percentage of single-headed households (reverse coded), and third-grade reading proficiency tended to be highly weighted.

Similar patterns in WQS weights were observed in sensitivity analyses of NDVI in varying buffer sizes (Supplemental Fig. 3). The magnitude of estimates attenuated with increasing buffer size for associations in the hypothesized direction and attenuated with decreasing buffer size for associations in the opposite direction.

### LASSO Regression

Including both individual and neighborhood characteristics in the model (Model C) only slightly reduced the MSE for NDVI and did not reduce the MSE for tree cover or park proximity compared to the model with only neighborhood-level predictors (Model A), suggesting that adding the individual-level predictors does not meaningfully improve prediction accuracy (Table 2). In prediction models for each of the three types of greenspace, the MSE was highest when only individual-level covariates were included in the model.

**Table 2:** Minimum mean squared error (MSE) from cross-validation of LASSO models of NDVI, tree cover, and park proximity

	NDVI	Tree cover	Park proximity
Model A: only neighborhood-level predictors^a^	0.005995	123.2869	511,426.9
Model B: only individual-level predictors^b^	0.007085	153.6273	682,977.9
Model C: both individual and neighborhood predictors^c^	0.005973	125.4239	534,456.0

^a^Model A includes the following measures at the census tract level: poverty rate, public assistance rate, homeownership rate, high-skill employment, median household income, employment rate, commute duration, single-headed households, school poverty, teacher experience, adult educational attainment, ECE centers, high-quality early childhood education centers, early childhood education enrollment, 3rd-grade math proficiency, 3rd-grade reading proficiency, high school graduation rate, advanced placement course enrollment, college enrollment in nearby institutions, racial composition (% Black/African-American), and racial residential segregation (*G* statistic)

^b^Model B includes the following measures, reported at the CANDLE age 4–6 study visit: maternal age, race, education, marital status, household income, and household size

^c^Model includes all predictors specified for models A and B

Supplemental Tables 1, 2, and 3 show coefficients from each model. In Model A (neighborhood-level), census tracts with lower proportions of Black residents and higher homeownership rate predicted higher NDVI. For tree cover, the largest coefficient was for the residential segregation *G* statistic, with a higher value indicating more residential segregation and predicting lower tree cover. Lower poverty rate and lower college enrollment nearby predicted both lower NDVI and lower tree coverage. In Model B (individual-level), marital status and maternal race predicted NDVI whereas maternal education predicted tree cover. Similar patterns were observed in Model C as in Model A for neighborhood-level predictors; more coefficients for individual-level variables were estimated to be non-zero in Model C, though the magnitude of these coefficients was small relative to those for neighborhood variables.

Smaller values of park proximity (i.e., living closer to the nearest park) were considered as greater availability of greenspace; education variables predicted park proximity across models A, B, and C, though in varying directions. Third grade reading proficiency, presence of ECE centers, and higher median household income predicted closer park proximity, but third grade math proficiency and high-quality ECE centers predicted a further distance from the nearest park, in Model A. In Model B, only two individual-level factors remained in the model, whereas almost all coefficients, including those at the individual level, remained in Model C.

## Discussion

In this study, we found that homeownership rate and early childhood education resources were associated with higher NDVI and tree cover. Homeownership was consistently weighted highly in the index of neighborhood factors, whereas multiple early education variables had smaller weights for each variable, but collectively suggest associations with the early educational environment. Furthermore, we observed disparities in residential greenness and tree cover for communities with a higher proportion of Black residents and a higher spatial measure of residential segregation. In contrast, models constrained in the opposite direction identified associations with lower greenspace when high school graduation rate, teacher experience, and single-headed households were weighted highly in the index. In prediction models, adding individual-level predictors to the model improved prediction of NDVI only slightly and did not improve prediction for trees or park proximity, though individual-level predictors were still frequently included in these combined models.

Prior literature has primarily focused on measures of income in relation to urban greenspace. Studies in Europe, Australia, and the US identified better access to greenspace in neighborhoods characterized by higher median incomes and lower concentration of poverty [17, 18, 42–45]. However, associations between income and park access are less clear [46]. Prior US studies have found more overall park access in low-income neighborhoods, but this relationship did not hold for access to safe parks or high-quality parks [47, 48]. We did not observe consistent results for greenness and tree canopy across measures of poverty and income; patterns of park access by income were inconsistent and appeared sensitive to modeling choices (e.g., in LASSO models, coefficients for neighborhood-level and individual-level income had opposite signs), though we were unable to account for park facilities for children such as playgrounds or park quality metrics. In WQS models of NDVI and tree canopy, median household income was less influential than housing tenure, though homeownership serves as an indicator of overall wealth, particularly among those with lower incomes in the US [49]. Others have also observed associations of housing tenure with greenness and suggested that renters may have less ability or incentive to influence green infrastructure [14, 50].

Fewer studies have considered education in addition to income in relation to greenspace. A multi-city study identified more consistent relationships between adult educational attainment and both greenness and woody vegetation, than those observed for income [5]. However, income had a larger role in cities with relatively lower per capita incomes and relationships between income, education, and urban vegetation were weak in the smallest cities in the study [5]. In our study, adult educational attainment was one of the top predictors of tree canopy when using neighborhood predictors in LASSO regression and maternal education was a top predictor in individual-level LASSO models, but adult educational attainment was not weighted heavily in WQS analyses. In contrast, early childhood education access figured prominently in many of our models; the weights for the proximity of ECE centers and elementary school test scores in WQS models in the hypothesized direction suggest a relationship with the early education environment. Given the high cost of early childhood education in the US, disparities in access to these education opportunities indicated by variables such as the percentage of 3- and 4-year-olds enrolled in preschool, likely reflect disparities in access to a broader set of resources for families with young children [51]. Some of these measures may reflect the population density of young children, which may vary relative to the total population density that we controlled for in this analysis, and has been correlated with measures of urban trees in other studies [52]. School grounds may also contribute to the quantity of greenspace in the neighborhood, particularly in high-income neighborhoods [53].

However, relationships between measures of the educational environment and greenspace were not consistent across all models in our study, limiting interpretation of these results. For example, high school graduation rate was highly weighted in WQS models in the opposite of the hypothesized direction. Additionally, early education and elementary education variables predicted park proximity in varying directions in LASSO models.

Differences in availability of greenspace by race and ethnicity have also been previously identified [12, 13]. Studies using measures of neighborhood racial composition have observed less greenspace availability in neighborhoods with a higher percentage of Black residents [54, 55]. We observed a similar pattern in our study and this disparity in greenspace exposures for neighborhoods with a higher proportion of Black residents was more pronounced for NDVI and tree cover relative to park proximity. Furthermore, in our descriptive analysis, census tracts with a higher proportion of Black residents were also negatively correlated with high socioeconomic and educational opportunity and high homeownership. Our results highlight the multiple ways in which structural racism leads to inequitable access to social and built environment resources to promote health and well-being.

Fewer studies of greenspace have used a spatial racial residential segregation measure [12, 56]. Though multiple approaches to assessing the different dimensions of racial residential segregation have been used, uncertainties remain as to the most useful measure in relation to the built environment. When using a spatial measure of residential segregation, the *G* statistic, we found higher residential segregation was the largest neighborhood coefficient predicting less tree canopy in LASSO models. Both the *G* statistic and the racial composition measure were weighted heavily in the WQS model of tree cover. Others have hypothesized that tree canopy in particular is more reflective of long-term investments in green infrastructure, given the time it takes for large trees to grow [14].

There are several limitations that should be considered in interpretation of these analyses. The overall levels of residential surrounding greenness and tree canopy in this sample were generally high with limited variability, as the regional climate is conducive to widespread vegetation growth and deciduous tree cover. Furthermore, the exposure metrics assessed are not mutually exclusive, as nearby green parks and tree canopy are captured in the NDVI assessment. The exposure measure also only assessed the quantity of greenspace, which does not account for the quality of or amenities in the greenspace, or access to other nature features such as proximity to water. Focus on greenspace around residences where children live may also limit generalizability to the full adult population. A second limitation of this analysis is that we used census tracts to define neighborhoods when assessing neighborhood resources and characteristics, as is commonly done in the literature, but these administrative boundaries may not accurately reflect the way that residents define or interact with their neighborhood. Additionally, these boundaries lead to a spatial mismatch between the neighborhood census tract variables and NDVI measures, which were measured within buffers around the residential locations irrespective of census geographical borders; this misalignment is a limitation in this analysis. An additional measurement limitation in this analysis is the use of racial composition and the spatial residential segregation measures derived from census tract racial composition, as these variables do not capture all the different components of structural racism. Our analysis provides a snapshot of only a single point in time, but future work could additionally consider changes over time, as greenspace has been linked to gentrification trends [57].

Some variability in our results was observed across methods. While some variables were influential in both modeling approaches (e.g., homeownership rate), we also observed some differences across the WQS and LASSO approaches. The WQS model tends to split weights across the group of variables that are highly correlated, while the LASSO model selects a single predictor out of a group of correlated predictors [38]. The differences in how these two modeling approaches handle correlated predictors may explain variability in the influence of predictors such as neighborhood poverty level across the various models. Furthermore, we observed associations between neighborhood measures and greenspace in the opposite of the hypothesized direction for all three greenspace measures in WQS regression, with different variables weighted heavily compared to models constrained to the hypothesized direction. Rapid development in this field includes new methods and further refinement of existing approaches that may further clarify these relationships, but a comprehensive comparison of results from additional mixtures approaches was beyond the scope of this paper.

This analysis is distinct from most prior studies examining relationships between socioeconomic factors and greenspace in that we used measures of greenspace in children’s immediate residential neighborhood. We explored multiple metrics of greenspace which may be of varying importance for a particular health behavior or health outcome of interest. We took a novel approach to exploring relationships between greenspace and other neighborhood features, by implementing a statistical method developed in the context of chemical mixtures. A further strength of this approach was the consideration of a wide selection of neighborhood characteristics and resources. We were able to include several commonly utilized economic indicators, as well as a spatial measure of racial residential segregation and a range of neighborhood factors related to educational opportunities that may be particularly relevant for child health and development.

This analysis highlights several variables for further investigation of greenspace disparities, including homeownership, early childhood education, and racial residential segregation. Our results further indicate the importance of accounting for confounding by neighborhood-level factors in analyses of greenspace and health, especially when neighborhood-level factors may be operating through multiple pathways to affect health. Associations with greenspace in the opposite of the hypothesized direction suggest careful consideration is needed in selecting potential neighborhood-level confounders. Given the array of health benefits hypothesized for exposure to greenspace, especially for children, the relationships of socioeconomic and educational variables, as well as residential segregation, with greenspace suggest that these neighborhood measures warrant consideration in developing policies and programs to improve equitable availability of greenspace.

### Supplementary Information

Below is the link to the electronic supplementary material.

Supplementary file1 (DOCX 551 KB)

## Data Availability

The data utilized for this study are not publicly available but de-identified data may be available on request, subject to approval by the internal review board and under a formal data use agreement. Contact the corresponding author for more information.
