# Supplementary material for: Individual and Neighborhood Level Predictors of Children’s Exposure to Residential Greenspace
Source: J Urban Health. 2024 Mar 14;101(2):349–63. doi: 10.1007/s11524-024-00829-z (PMC11052952; doi:10.1007/s11524-024-00829-z)
Supplement: Supplementary file 1 — Supplementary file1 (DOCX 551 KB) [file 11524_2024_829_MOESM1_ESM.docx]

**Supplementary Material**

**Individual and neighborhood level predictors of children’s exposure to residential greenspace**

Marnie F. Hazlehurst, Anjum Hajat, Adam A. Szpiro, Pooja S. Tandon, Joel D. Kaufman, Christine T. Loftus, Nicole R. Bush, Kaja Z. LeWinn, Marion E. Hare, Sheela Sathyanarayana, Catherine J. Karr

**Contents**

**Appendix A.** Selection of analytic approach.

**Figure 1.** Shelby County census tracts with CANDLE participants.

**Figure 2.** Spearman correlations among variables characterizing multiple domains of the neighborhood environment.

**Figure 3.** Sensitivity analysis of NDVI in varying buffer sizes.

**Table 1.** Standardized coefficients from the LASSO models using only neighborhood-level predictors (Model A).

**Table 2.** Standardized coefficients from LASSO models using only individual-level predictors (Model B)**.**

**Table 3.** Standardized coefficients from LASSO models including predictors at both the neighborhood and individual levels (Model C).

**Appendix A.** Selection of analytic approach.

There are multiple methods that can potentially be used in examining the mixture of neighborhood factors considered in our analysis. These methods for examining mixtures have developed rapidly in recent years, including but not limited to Quantile G-Computation, Bayesian Index Model, Bayesian Kernel Machine Regression (BKMR), and Weighted Quantile Sum (WQS) regression. Each method has several strengths and limitations and each method has utility in answering slightly different research questions. An extensive discussion of the merits of the various approaches has been published in the literature [1–8]. We briefly summarize the approaches here.

Quantile G-Computation estimates the overall effect of a mixture by summing the corresponding regression coefficients [1]. Thus, counteracting positive and negative effects may effectively cancel one another out when estimating the aggregate effect. However, there may be scenarios in which experiencing both exposures does not have the same effect as experiencing neither of the exposures. For example, associations between coffee, alcohol, and alertness have been cited as an example where each exposure (coffee and alcohol) has an association with the outcome (alertness) but in opposite directions [2]. In this case, quantile g-computation estimates no overall effect though the biological effects of experiencing both exposures is different than experiencing neither exposure.

The Bayesian Grouped Index Model (BGIM) allows for different subsets of the mixture to have effects opposite directions (e.g. herbicides and insecticides were identified to have associations with childhood leukemia risk in different directions) [7]. The Bayesian index model was also developed to address the two-step process of fitting WQS regression models and simulations further suggest the Bayesian index model performs better in certain settings with small (<500) sample sizes than WQS [7]. However, this method requires prespecifying the groups within the mixture, limiting utility in this case where we do not have such clearly defined subgroups.

BKMR estimates an overall effect allowing for nonlinear effects and interactions between mixture components [8]. However, BKMR requires a larger sample size and is more computationally intensive than other methods.

WQS regression aims to estimate an overall effect as well as to identify the ‘toxic agents’ in the mixture based on the weights in the index [9, 10]. In contrast to quantile g-computation, WQS explicitly allows us to disentangle potential associations with the outcome in both positive and negative directions with varying weights in the mixture, and unlike BGIM it does not require prespecifying which mixture components will act in the same direction. A limitation of the standard implementations of WQS regression is that it requires both training and test data, as applying the two-step procedure in the full sample is known to produce p-values that are anti-conservative. However, a permutation test has been developed to address this issue when using WQS in the full sample [4].

After careful consideration of the strengths and limitations of an array of mixture methods as briefly described above, we consider WQS to be the most appropriate approach for this analysis because we are interested in potential associations in each direction, we do not have *a priori* hypotheses about which mixture components will act in the same direction, and we are able to address the limitation of anti-conservative p-values in WQS by implementing the permutation test.

**References**

1. Keil AP, Buckley JP, O’Brien KM, Ferguson KK, Zhao S, White AJ (2020) A Quantile-Based g-Computation Approach to Addressing the Effects of Exposure Mixtures. Environ Health Perspect 128:047004. https://doi.org/10.1289/EHP5838

2. Gennings C (2021) Comment on “A Quantile-Based g-Computation Approach to Addressing the Effects of Exposure Mixtures.” Environ Health Perspect 129:038001. https://doi.org/10.1289/EHP8739

3. Keil AP, Buckley JP, O’Brien KM, Ferguson KK, Zhao S, White AJ (2021) Response to “Comment on ‘A Quantile-Based g-Computation Approach to Addressing the Effects of Exposure Mixtures.’” Environ Health Perspect 129:038002. https://doi.org/10.1289/EHP8820

4. Day DB, Sathyanarayana S, LeWinn KZ, Karr CJ, Mason WA, Szpiro AA (2022) A Permutation Test-Based Approach to Strengthening Inference on the Effects of Environmental Mixtures: Comparison between Single-Index Analytic Methods. Environ Health Perspect 130:087010. https://doi.org/10.1289/EHP10570

5. Keil AP, Buckley JP, O’Brien KM, Ferguson KK, White AJ (2023) Comment on “A Permutation Test-Based Approach to Strengthening Inference on the Effects of Environmental Mixtures: Comparison between Single-Index Analytic Methods.” Environ Health Perspect 131:018001. https://doi.org/10.1289/EHP12404

6. Day DB, Sathyanarayana S, LeWinn KZ, Karr CJ, Mason WA, Szpiro AA (2023) Response to “Comment on ‘A Permutation Test-Based Approach to Strengthening Inference on the Effects of Environmental Mixtures: Comparison between Single-Index Analytic Methods.’” Environ Health Perspect 131:018002. https://doi.org/10.1289/EHP12517

7. Wheeler DC, Rustom S, Carli M, Whitehead TP, Ward MH, Metayer C (2021) Bayesian Group Index Regression for Modeling Chemical Mixtures and Cancer Risk. IJERPH 18:3486. https://doi.org/10.3390/ijerph18073486

8. Bobb JF, Valeri L, Claus Henn B, Christiani DC, Wright RO, Mazumdar M, Godleski JJ, Coull BA (2015) Bayesian kernel machine regression for estimating the health effects of multi-pollutant mixtures. Biostatistics 16:493–508. https://doi.org/10.1093/biostatistics/kxu058

9. Carrico C, Gennings C, Wheeler DC, Factor-Litvak P (2015) Characterization of Weighted Quantile Sum Regression for Highly Correlated Data in a Risk Analysis Setting. JABES 20:100–120. https://doi.org/10.1007/s13253-014-0180-3

10. Czarnota J, Gennings C, Wheeler DC (2015) Assessment of Weighted Quantile Sum Regression for Modeling Chemical Mixtures and Cancer Risk. Cancer Inform 14s2:CIN.S17295. https://doi.org/10.4137/CIN.S17295

**Figure 1.** Shelby County census tracts with CANDLE participants.


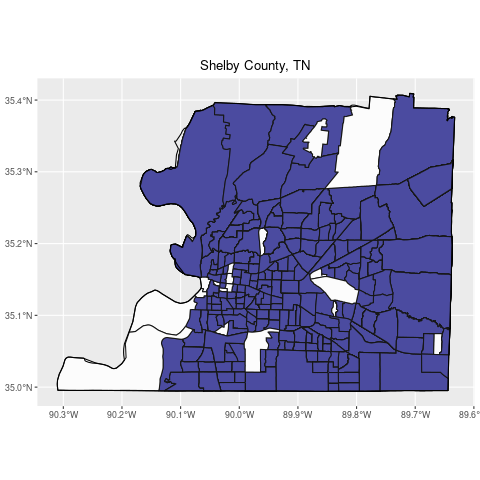


**Figure 2.** Spearman correlations among variables characterizing multiple domains of the neighborhood environment.


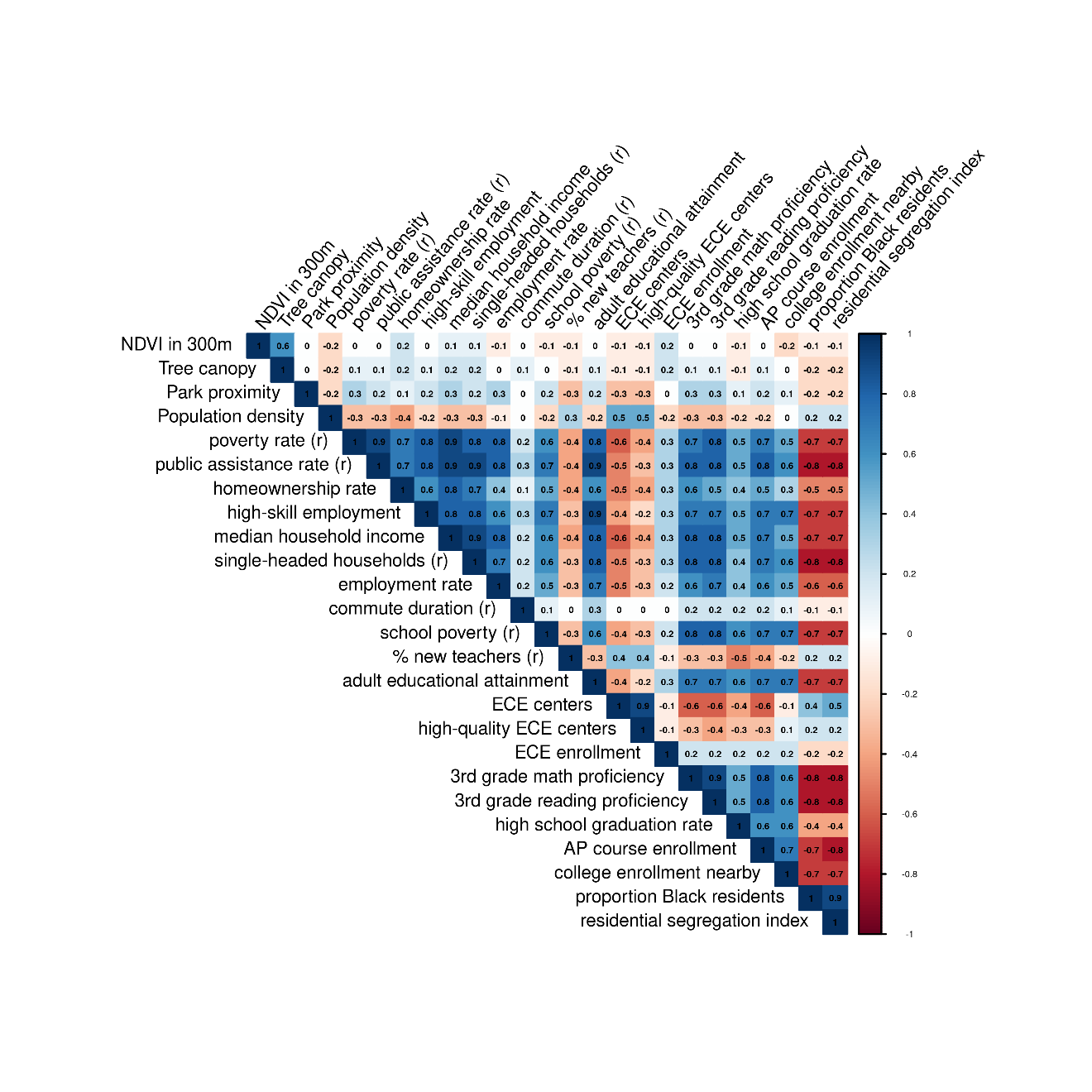


**Figure 3.** Sensitivity analysis of NDVI in varying buffer sizes.


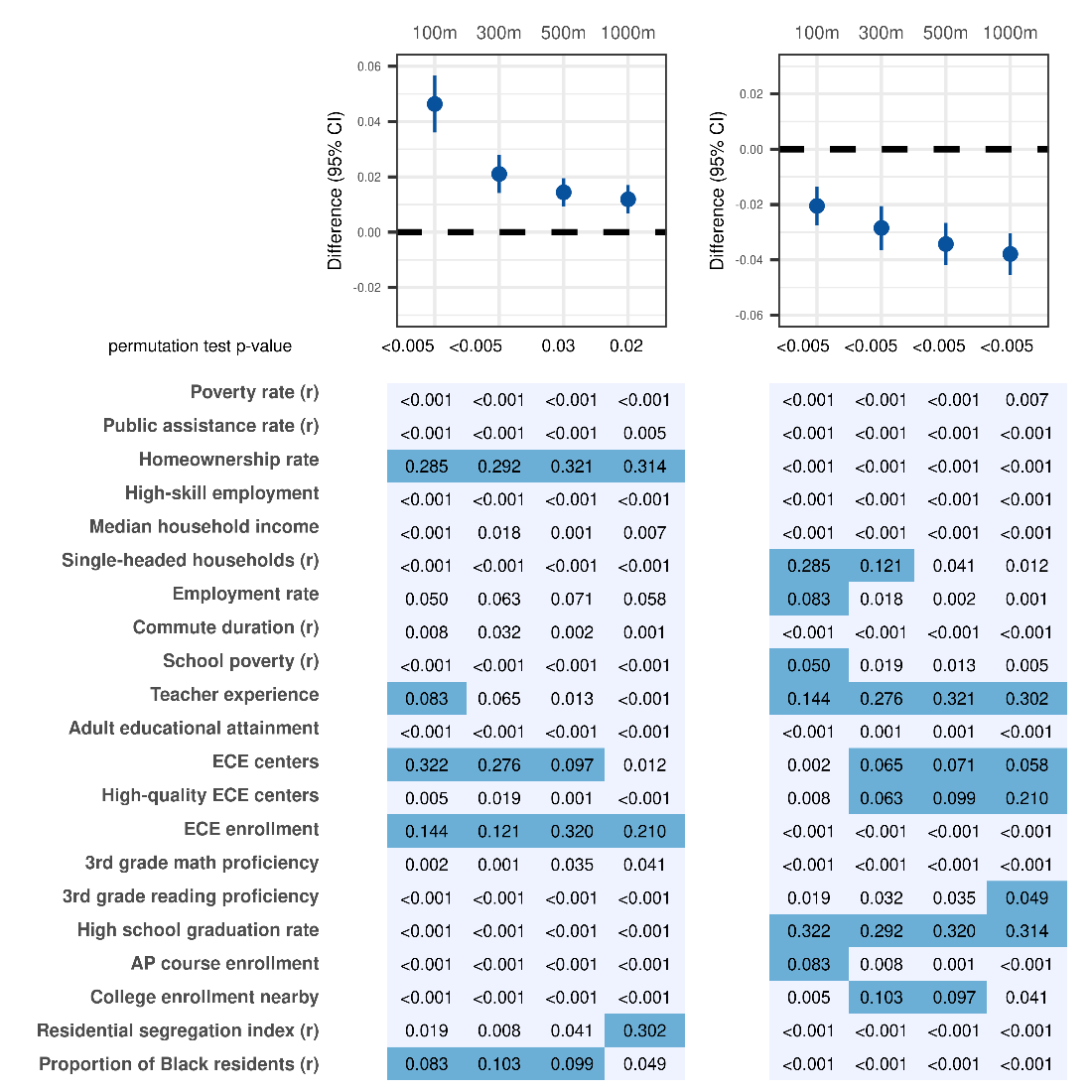


Plots show the difference (95% confidence interval) in surrounding NDVI in various buffer sizes per 1 quintile higher WQS index and corresponding weights, with the p-value from the permutation test displayed underneath. Results from models constrained in the positive direction are shown on the left and results from the model constrained in the negative direction are shown on the right. The buffer size of the NDVI measure for each column of results is indicated at the top of each column of results, ranging from 100m to 1000m. The darker shade of blue indicates weights >0.048 in the WQS index; this cutoff value would be the value of all weights in the index if each variable in the index was weighted equally. Some variables were reverse coded, indicated by (r), so that variables contributing to the WQS index were coded such that a higher value of the variable was hypothesized to be associated with more greenspace. In the model constrained in the positive direction, associations were largely driven by homeownership rate across all buffer sizes. High school graduation rate and teacher experience were weighted highly in the model constrained in the negative direction.

**Table 1.** Standardized coefficients from the LASSO models using only neighborhood-level predictors (Model A).

| **Model A Predictors** | **NDVI** ^a, b^ | **Tree Canopy** ^a, b^ | **Park Proximity** ^a, c^ |
| --- | --- | --- | --- |
| **Neighborhood-level ^d^** |  |  |  |
| Poverty rate (r) | -0.024 | -4.91 | 179.6 |
| Public assistance rate (r) | -0.007 | -0.54 | 29.3 |
| Homeownership rate | 0.028 | 3.07 | -83.7 |
| High-skill employment | -0.002 | 0.24 | 28.4 |
| Median household income | 0.004 | 0.64 | -263.3 |
| Employment rate |  |  | -95.7 |
| Commute duration (r) | 0.004 | 0.74 | -26.3 |
| Single-headed households (r) |  | 1.65 | 116.0 |
| School poverty (r) | -0.004 | -1.25 | -71.8 |
| % new teachers (r) | -0.009 |  | -168.6 |
| Adult educational attainment |  | 2.25 | 90.5 |
| Early childhood education centers | -0.004 | 0.49 | -637.2 |
| High-quality early childhood education centers | -0.002 | 0.36 | 259.9 |
| Early childhood education enrollment | 0.010 | 1.20 | -50.2 |
| Third grade math proficiency |  |  | 724.2 |
| Third grade reading proficiency | -0.006 | -0.50 | -911.4 |
| High school graduation rate |  |  | -69.3 |
| Advanced Placement course enrollment | -0.013 | -0.76 | 60.0 |
| College enrollment in nearby institutions | -0.024 | -4.89 | 63.7 |
| Proportion Black/African-American residents | -0.039 | -0.69 | 237.9 |
| Residential segregation index (G-statistic) | -0.005 | -6.09 | -247.3 |

^a^ Reported coefficients are from models using the tuning parameter identified by the minimum MSE from 10-fold cross-validation. Blank cells indicate variables where coefficients were estimated to be zero in the LASSO model. ^b^ For NDVI and tree canopy models, a higher value represents more green space. ^c^ In contrast, the parks measure is operationalized as distance to the nearest park (in meters) with smaller values being interpreted as greater access to green space. ^d^ Neighborhood-level variables are included in the model as z-scores that were then standardized to mean of zero and standard deviation of 1.

**Table 2.** Standardized coefficients from LASSO models using only individual-level predictors (Model B). ^a^

| **Model B Predictors** | **NDVI** ^b^ | **Tree Canopy** ^b^ | **Park Proximity** ^c^ |
| --- | --- | --- | --- |
| **Individual-level** |  |  |  |
| Maternal race: Black/African-American | -0.013 | -1.46 |  |
| Maternal education: High school degree |  |  | 62.3 |
| Maternal education: technical school |  | -1.32 |  |
| Maternal education: college degree |  |  |  |
| Maternal education: graduate/professional degree |  | 2.46 |  |
| Income | 0.0004 | 0.73 | 95.0 |
| Household size | 0.003 | 0.67 |  |
| Married/living with partner | 0.012 | 1.59 |  |
| Maternal age |  | 0.05 |  |

^a^ Blank cells indicate variables where coefficients were estimated to be zero in the LASSO model. Reported coefficients are from models using the specified tuning parameter identified by the minimum MSE from 10-fold cross-validation. ^b^ For NDVI and tree canopy models, a higher value represents more green space. ^c^ In contrast, the parks measure is operationalized as distance to the nearest park (in meters) with smaller values being interpreted as greater access to green space.

**Table 3.** Standardized coefficients from LASSO models including predictors at both the neighborhood and individual levels (Model C).

| **Model C Predictors** | **NDVI ^a, b^** | **Tree Canopy ^a, b^** | **Park Proximity ^a, c^** |
| --- | --- | --- | --- |
| **Neighborhood-level ^d^** |  |  |  |
| Poverty rate (r) | -0.023 | -4.93 | 158 |
| Public assistance rate (r) | -0.007 | -0.53 | 8 |
| Homeownership rate | 0.026 | 2.87 | -61 |
| High-skill employment | -0.003 | 0.52 | 34 |
| Median household income | 0.002 | 0.56 | -244 |
| Employment rate |  |  | -70 |
| Commute duration (r) | 0.004 | 0.82 | -21 |
| Single-headed households (r) | 0.000 | 1.76 | 99 |
| School poverty (r) | -0.003 | -1.22 | -72 |
| % new teachers (r) | -0.009 |  | -163 |
| Adult educational attainment |  | 1.81 | 69 |
| Early childhood education centers | -0.005 | 0.29 | -611 |
| High-quality early childhood education centers | -0.002 | 0.40 | 248 |
| Early childhood education enrollment | 0.010 | 1.23 | -51 |
| Third grade math proficiency |  |  | 556 |
| Third grade reading proficiency | -0.009 | -1.04 | -726 |
| High school graduation rate |  | -0.05 | -47 |
| Advanced Placement course enrollment | -0.013 | -0.75 | 51 |
| College enrollment in nearby institutions | -0.025 | -5.03 | 60 |
| Proportion Black/African-American residents | -0.036 | -0.66 | 192 |
| Residential segregation index (G-statistic) | -0.006 | -6.21 | -233 |
| **Individual-level predictors** |  |  |  |
| Maternal race: Black/African-American | -0.010 | 0.35 | 32 |
| Maternal education: High school degree | -0.004 | 0.02 | 69 |
| Maternal education: technical school degree | -0.008 | -1.70 | 96 |
| Maternal education: college degree | -0.004 | 1.32 |  |
| Maternal education: graduate/professional degree |  |  |  |
| Household income | 0.005 | 0.76 | -2 |
| Household size | 0.005 | 0.92 | 3 |
| Marital status: married/living with partner | 0.005 | 0.82 | 26 |
| Maternal age | -0.001 | -0.25 |  |

^a^ Reported coefficients are from models using the specified tuning parameter identified by the minimum MSE from 10-fold cross-validation. Blank cells indicate variables where coefficients were estimated to be zero in the LASSO model. ^b^ For NDVI and tree canopy models, a higher value represents more green space. ^c^ In contrast, the parks measure is operationalized as distance to the nearest park (in meters) with smaller values being interpreted as greater access to green space. ^d^ Neighborhood-level variables are included in the model as z-scores that were then standardized to mean of zero and standard deviation of 1.
